# Influence of Both La Nina and Island Isolation During COVID-19 on the Epidemiology of Infectious Diseases in New Caledonia

**DOI:** 10.3390/epidemiologia7030070

**Published:** 2026-05-21

**Authors:** Pierre-Henri Moury, Ann-Claire Gourinat, Maria Suveges, Méryl Delrieu, Myrielle Dupont-Rouzeyrol, Christophe Menkes, Nathanaëlle Soler, Cécile Cazorla, Antoine Biron, Antoine Flahault, Morgan Mangeas, Nicolas Ray

**Affiliations:** 1 MISIT (Méthodologie de l’Information en Santé, Biostatistiques, Innovations Technologiques), Pôle de Santé Public, Centre Hospitalier Universitaire Grenoble Alpes, TIMC (Recherche Translationnelle et Innovation en Médecine et Complexité), Centre Investigation Clinique-Innovation Technologique, Université Grenoble Alpes, 38000 Grenoble, France; 2 Centre Hospitalier Territorial Gaston-Bourret, 98835 Dumbea-Sur-Mer, New Caledonia, France; acgourinat@hotmail.com (A.-C.G.); cecile.cazorla@cht.nc (C.C.); antoine.biron@cht.nc (A.B.); 3 Institute of Global Health, Campus Biotech, University of Geneva, 1202 Geneva, Switzerland; maria.suveges@unige.ch (M.S.); antoine.flahault@unige.ch (A.F.); nicolas.ray@unige.ch (N.R.); 4 Institut Pasteur de Nouvelle-Caledonie, URE Dengue et Arboviroses, 98845 Noumea, New Caledonia, France; mdelrieu@pasteur.nc (M.D.); mdupont@pasteur.nc (M.D.-R.); 5 ENTROPIE, Institut de Recherche pour le Développement (IRD), Centre National de la Recherche Scientifique (CNRS), Institut Français de Recherche Pour L’Exploitation de la Mer (IFREMER), Université de la Nouvelle-Calédonie, 98800 Noumea CEDEX, New Caledonia, France; christophe.menkes@ird.fr (C.M.); morgan.mangeas@ird.fr (M.M.); 6 EMR9001 SantEco, CNRS, IRD, University of New Caledonia, 98848 Noumea CEDEX, New Caledonia, France; 7 Centre Population et Développement, IRD, 98848 Noumea CEDEX, New Caledonia, France; nathanaelle.soler@ird.fr; 8 Institute for Environmental Sciences, University of Geneva, 1205 Geneva, Switzerland

**Keywords:** emergent infectious diseases, water-borne diseases, vector-borne disease, travel, leptospirosis, dengue, influenza, El Niño/Southern Oscillation, ENSO

## Abstract

*Background and Objectives*: New Caledonia, an archipelago in the South Pacific, experienced an unprecedented conjunction of prolonged border closure during the COVID-19 pandemic (2020 to 2022) and marked influence of the El Niño/Southern Oscillation (ENSO). This context provided a unique opportunity to explore how environmental drivers, island isolation, and socio-demographic factors interact to shape infectious disease dynamics. This study aimed to assess the respective and combined effects of climatic variability, travel restrictions, and socio-demographic factors on the dynamics of four priority infectious diseases. *Materials and Methods*: We retrospectively analysed data from 2017 to 2023 on four infectious diseases: leptospirosis, dengue, influenza, and hepatitis A (HAV). Satellite precipitation data and the Multivariate El Niño/Southern Oscillation Index (MEI) were used. Socio-demographic and economic variables were gathered. Statistical analyses employed descriptive analysis and Generalized Additive Mixed Models to evaluate the associations between climatic events, travel restrictions, and disease circulation using the communal level as a random effect and time (daily) as a spline effect. *Results*: We analysed 878 cases of leptospirosis, 165 of HAV, 6607 of influenza, and 7377 dengue cases. Influenza was associated with rainfall before lockdown (Odds Ratio (OR) 0.7, Confidence interval 95%, (CI95%), (0.6–0.8)) and disappeared during lockdown but resurged post-reopening losing its meteorological association. Dengue epidemics declined, coinciding with the *Wolbachia* program and border closure, and were associated with lower MEI (OR 0.78, CI95% (0.6–1) during the 2017 to 2020 period. HAV cases were correlated with the MEI (OR: 1.8, CI95% (1–3.3)). Leptospirosis cases were associated with cumulative rainfall (OR 1.12 (1.1–1.2)) and lower education (OR 1.04, CI95% (1–1.1)) and decreased with water supply (OR 0.7, CI95% (0.5–0.8)). *Conclusions*: Our findings highlight how climatic conditions, mobility restrictions, and socio-environmental inequities differentially shape infectious disease risks in island ecosystems. These results reinforce the need for integrated One Health surveillance that jointly addresses environmental change, social vulnerability, and infectious disease prevention.

## 1. Introduction

New Caledonia, a French archipelago in the South Pacific, is a biodiversity hotspot and is highly vulnerable to climate change. Its unique geographical position and colonial history have shaped a diverse population and contributed to pronounced sociodemographic vulnerabilities [1,2,3].

During the COVID-19 pandemic, New Caledonia successfully implemented a Zero-COVID policy, with two lockdowns: the first in March 2020 [4,5] and the second in March 2021 [6] (see Supplementary Materials for a detailed description of the period). In September 2021, a breach associated with the Delta variant led to a surge in cases, progressively bringing strict border controls to an end by December 2021 [7].

Concurrently, weather conditions influenced by the El Niño–Southern Oscillation (ENSO) shifted markedly between 2020 and 2022, with the onset of La Niña conditions characterized by heavy rainfall and increased temperatures [8,9], coinciding with the period of border closure. La Niña phases generally favour above-normal rainfall over New Caledonia, particularly during the austral warm season, through the southwestward extension and intensification of the South Pacific Convergence Zone; however, the magnitude and spatial expression of these anomalies vary by season and decade [8,9]. Therefore, this period represents a unique opportunity to investigate the influence of a major climatic phenomenon such as ENSO on infectious disease circulation in the absence of international travel.

We aimed to analyse the dynamics of infectious diseases during archipelago-wide border closure. Four infectious diseases were selected based on their relevance in this specific setting, as documented in the literature: dengue [10,11,12], influenza [13], hepatitis A [14], and leptospirosis [15,16]. Each disease exhibits distinct transmission patterns linked to its specific epidemiological characteristics, making them valuable indicators for assessing particular risks during border closure. These diseases are known to be associated with either population mobility or environmental changes, both of which were substantially affected by the lockdown measures and concurrent climatic events [17,18]. A detailed description of each disease in the global and New Caledonian contexts is provided in the Supplementary Materials.

We hypothesized that climatic events such as ENSO were associated with infectious disease dynamics independently of international travel. In New Caledonia, rainfall serves as a reliable proxy for La Niña events [19]. ENSO phases were characterized using the Multivariate El Niño/Southern Oscillation Index (MEI).

First, we analyzed temporal and spatial trends in infectious disease incidence at the community level in relation to precipitation patterns and the MEI. Second, we assessed the impact of the territory-wide lockdown on infectious disease circulation. Finally, we examined post-reopening incidence trends following the lifting of the travel ban.

## 2. Materials and Methods

This retrospective database study was conducted in accordance with the principles of the Declaration of Helsinki and was approved in 2021 by the research board of the Centre Hospitalier Territorial Gaston-Bourret de Nouvelle-Calédonie (CHT). The study was designed to include data retrospectively at the end of 2023.

### 2.1. Study Location, Communities and Historical Setting

New Caledonia is an archipelago in the South Pacific with a high degree of autonomy within the French Republic. The main island is characterised by a central mountain range that creates marked climatic contrasts, with higher rainfall on the east coast and drier conditions on the west coast.

With a population of approximately 270,000 during the study period, New Caledonia is home to the Indigenous Kanak people as well as other groups resulting from successive waves of migration during the colonial era, including Europeans and communities from former Southeast Asian and Pacific colonies. These populations are unevenly distributed across the territory, with the majority concentrated in Nouméa, the capital. As a legacy of settler colonialism, the European population is primarily concentrated in Nouméa and the Southern Province, whereas the Northern Province and the Loyalty Islands are predominantly inhabited by Kanak communities.

Border closure measures in New Caledonia began on 20 March 2020 and ended in December 2021 [1,4,7] (See Supplementary Materials for a more detailed description of the period). During this period, three lockdowns took place in response to COVID-19 introductions. The Zero-COVID strategy involved two strict lockdowns: the first started on 23 March 2020 [4,5], and the second on 9 March 2021 [6]. In September 2021, a breach associated with the Delta variant led to a surge in cases, rendering strict border controls unnecessary by the end of December 2021 [7].

### 2.2. Data Collection

#### 2.2.1. Meteorological Variables

Satellite-derived precipitation data (MSWEP, https://www.gloh2o.org/mswep/ (accessed on 14 of October 2024)) were used to estimate mean monthly rainfall from 1 January 2017 to 31 December 2023. These data were graphically compared with observations from the New Caledonia meteorological stations (https://www.data.gouv.fr/fr/datasets/donnees-climatologiques-de-base-quotidiennes/ (accessed on 14 of October 2024)) to assess the need for potential correction.

El Niño/Southern Oscillation (ENSO) periods were identified using reports from the Australian Government Bureau of Meteorology (http://www.bom.gov.au/climate/enso/wrap-up/archive.shtml (accessed on 14 of October 2024), based on the bi-monthly Multivariate ENSO Index (MEI). The MEI (https://psl.noaa.gov/enso/mei/ (accessed on 14 of October 2024)) is a time series that integrates five variables—sea level pressure, sea surface temperature, zonal and meridional surface wind components, and outgoing longwave radiation—across the tropical Pacific basin (30° S–30° N, 100° E–70° W), enabling the classification of El Niño, La Niña, and neutral phases within a single composite index [20].

Rolling monthly averages of the MEI and precipitation were linearly interpolated to obtain daily values. To investigate associations with epidemic outbreaks, we calculated the mean precipitation for each communal district over one-month periods.

Only the MEI and satellite-derived precipitation were included in the final models, although other climatic variables—such as temperature and relative humidity—were available. This selection was guided primarily by our objective of characterising overall (macroscopic) dynamics, given that heavy rainfall and La Niña conditions were considered the principal climatic drivers of both environmental change and the infectious diseases under study. In addition, exploratory analyses indicated substantial collinearity between precipitation and humidity or temperature (see Supplementary Materials). In this subtropical island setting, relative humidity and temperature appeared to be more strongly influenced by local characteristics within communal districts. Because ENSO directly influences rainfall patterns, we chose to include only one of these correlated variables in the final model and to exclude temperature and relative humidity due to collinearity with the MEI and precipitation.

#### 2.2.2. Infectious Diseases Report and Wolbachia Program Reporting

Cases of influenza, dengue, hepatitis A, and leptospirosis recorded between 2017 and 2023 were extracted from the database of the Centre Hospitalier Territorial (CHT), which compiles all notified cases across the territory for the healthcare and social services department. The four infectious diseases are further described in the Supplementary Materials, along with vaccination strategies when available. Cases were geographically assigned to one of the 33 communal districts of New Caledonia using the social insurance postal code and the declared residential address. When this information was missing, cases were excluded from the geographic analyses. All suspected cases of leptospirosis, dengue, and hepatitis A virus (HAV) infection were laboratory-confirmed by serology or polymerase chain reaction (PCR). Influenza cases were identified either through syndromic surveillance within the sentinel network or by biological diagnosis at the CHT laboratory; only biologically confirmed cases were included in the present analysis. For statistical analyses, we used the monthly number of reported cases and the incidence rate per 100,000 inhabitants, calculated as a one-month moving average for each communal district.

The *Wolbachia* program was included for descriptive and representational purposes (see Supplementary Materials) [21]. The World Mosquito Program^TM^ was launched in Nouméa in July 2019 and subsequently extended to surrounding suburban areas between 2022 and 2023 (https://www.worldmosquitoprogram.org/wmp-en-nouvelle-caledonie (accessed on 20 of November 2024)). This intervention involved the release of *Aedes aegypti* mosquitoes infected with *Wolbachia* to reduce the burden of vector-borne diseases. *Wolbachia* is a Gram-negative bacterium primarily transmitted vertically and is negatively associated with arbovirus transmission. It can manipulate host reproductive mechanisms to enhance maternal transmission through eggs. Owing to its ability to spread within mosquito populations and to reduce viral transmission, *Wolbachia* has been deployed as a biocontrol strategy to prevent arboviral diseases.

#### 2.2.3. Socio-Demographic and Economic Variables

Data from the 2019 census of New Caledonia provided information on demographic, social, and economic characteristics (https://www.isee.nc/publications/la-nouvelle-caledonie-en-cartes-et-en-chiffres/donnees-du-recensement-2019-en-open-data (accessed on 20 of February 2024)). Communal-level data were obtained from https://www.isee.nc/publications/la-nouvelle-caledonie-en-cartes-et-en-chiffres/cartographie-dynamique (accessed on 20 of February 2024). The spatial distribution of healthcare facilities was derived from 2024 publications of the Government of New Caledonia (https://nouvelle-caledonie.opendatasoft.com/explore/dataset/situation_etablissements_sante/ (accessed on 20 of February 2024)). In light of the differing pathogenesis of the diseases under study and the results of exploratory analyses, the following variables were retained: percentage of inhabitants using walking as their primary mode of transport; percentage of households with access to a water supply; percentage of inhabitants with a BEPC (lower secondary diploma) or lower level of education; and number of healthcare facilities per inhabitant.

### 2.3. Statistical Analysis

Statistical analyses were performed using R software (version 4.3.3; R Foundation for Statistical Computing) and QGIS (version 3.34). Categorical variables were expressed as counts (percentages), and comparisons were conducted using the chi-squared test or Fisher’s exact test, as appropriate. Continuous variables were reported as means with 95% confidence intervals (95% CI), and normality was assessed using the Shapiro–Wilk test. A two-sided *p*-value < 0.05 was considered statistically significant.

Principal component analysis (PCA) was conducted to explore socio-demographic and economic variables derived from the 2019 census. Variables were selected based on their variance and their relevance to disease pathogenesis (see Supplementary Figure S2).

To examine associations between climatic factors and infectious disease incidence, we used Generalized Additive Mixed Models (GAMMs). The baseline model included satellite-derived precipitation (monthly averages interpolated to daily values for each communal district), the Multivariate ENSO Index (MEI), time (modelled as a spline term), and communal district as a random effect. Time was incorporated as a penalized spline to flexibly capture non-linear temporal trends, thereby accounting for long-term trends and seasonality without imposing a predefined functional form (see Supplementary Materials for further details). The determination of lag structures is described in the Supplementary Materials.

GAMMs were implemented when the number of cases was sufficient across an adequate number of communal districts to allow stable estimation of random effects and to avoid overdispersion. Cases also had to be reliably linked to a residential address to minimise spatial misclassification. When case counts or spatial coverage were insufficient to support mixed modelling, a simpler Generalized Additive Model (GAM) without random effects was fitted at the territorial level to ensure model stability and reduce the risk of overdispersion or spatial bias.

Incidence distributions were assumed to follow a Tweedie distribution, a member of the exponential dispersion family suitable for zero-inflated data [22]. The Tweedie distribution accommodates a broad range of skewness and covariance structures for discrete and continuous longitudinal data [23]. In mixed models, the communal district was specified as a random effect. Variable selection was performed using backward stepwise elimination, guided by pathophysiological relevance, statistical significance (*p* < 0.05), and the Akaike Information Criterion (AIC). Final model coefficients were exponentiated and reported as odds ratios (ORs).

## 3. Results

**Figure 1:**
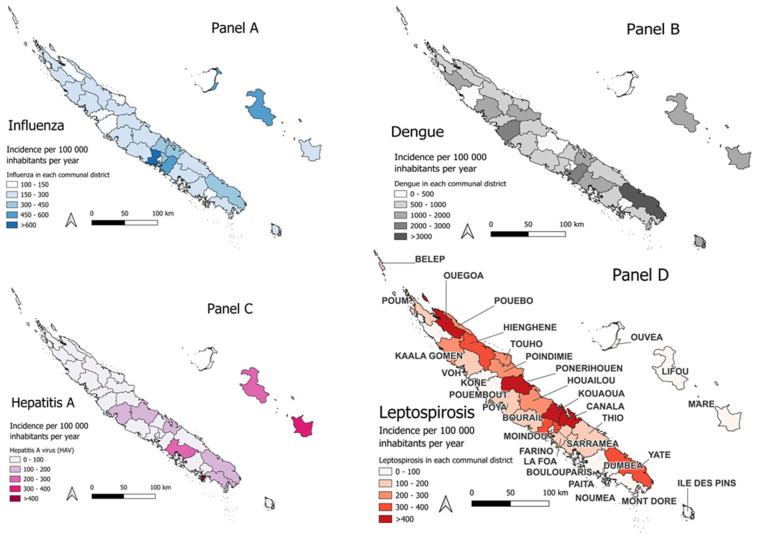
Spatial distribution of influenza, hepatitis A virus, dengue and leptospirosis in New Caledonia, at the communal district level and averaged over the 2017–2023 period. (**Panel A**) Influenza (biologically confirmed), (**Panel B**) dengue, (**Panel C**) hepatitis A virus, and (**Panel D**) leptospirosis. Map in (**D**) shows communal district names.

Overall, from 2017 to 2023, 15,024 positive samples were analysed. A total of 878 cases of leptospirosis, 165 cases of hepatitis A, and 7377 cases of dengue were identified. Among 6607 reported cases of influenza, 2022 were biologically confirmed (see Figure 1 and Table 1).

### 3.1. Meteorological and Socio-Demographic Variables

Rainfall and temperature increased following the first lockdown on 20 March 2020 (see Supplementary Materials, Supplementary Figure S1A,B). The Multivariate ENSO Index (MEI) indicated a shift towards La Niña conditions during the period of island-wide lockdown (Supplementary Figure S1C).

Socio-economic variables included in the analysis were initially explored using principal component analysis (PCA) (Supplementary Figure S2). The variables explaining the greatest proportion of variance reflected a socio-economic gradient, level of education, and whether inhabitants resided in tribal areas. Based on these results, the following variables were retained for further analysis: percentage of inhabitants using walking as their primary mode of transport; percentage of households with access to a water supply; percentage of inhabitants with a BEPC (lower secondary diploma) or lower level of education; and number of healthcare facilities per inhabitant.

### 3.2. Infectious Agents

The number of cases and the annual incidence per 100,000 inhabitants are presented in Table 1. The spatial distribution of incidence rates is shown in Figure 1. Table 2 summarizes the final models derived from the GAMM analyses, while Supplementary Table S1 provides the full models prior to stepwise variable selection. Supplementary Figure S3 illustrates the fitted prediction models in relation to the observed case distributions.

#### 3.2.1. Influenza

**Figure 2:**
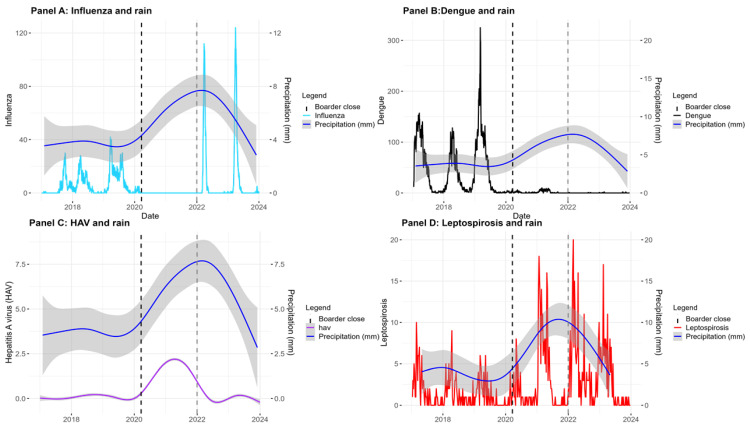
Evolution of cases and moving 7-day averages of influenza, hepatitis A virus HAV, dengue and leptospirosis with precipitation over New Caledonia. (**Panel A**), Influenza cases biologically confirmed and the 7-day rolling average of the cases (light blue). (**Panel B**), Dengue (black) and rain evolution. (**Panel C**), Hepatitis A virus (HAV), the first cases were diagnosed in 2018 (Purple). (**Panel D**), leptospirosis cases in red and the precipitation. Precipitation was shown in blue in each graph. The black dash line shows the Islands closure on 20 March 2020 and the grey dash line shows the opening of New Caledonia in December 2022.

Seasonal influenza epidemics followed a wave-like pattern between 2017 and 2020, which disappeared completely during the national lockdown period (2020–2021). Only biologically confirmed cases were included in the analysis. Following the reopening of the territory in December 2021, influenza activity resumed, with a steeper increase in epidemic waves observed in 2022 and 2023 (Figure 2A).

Given this marked disruption in epidemic dynamics, modelling was conducted excluding the post-reopening period. Using a GAMM analysis restricted to the pre-reopening years, higher rainfall was significantly associated with a lower incidence of influenza (OR: 0.7; 95% CI: 0.6–0.8; *p* < 0.0001). In contrast, after border reopening, influenza incidence appeared to be primarily driven by random variation, with no significant association with rainfall observed (Table 2).

#### 3.2.2. Dengue

The spatial distribution of dengue incidence is presented in Figure 1B, which illustrates the annual incidence rate per 100,000 inhabitants across communal districts during the study period (2017–2020). Temporal trends are shown in Figure 2B. A one-month lag provided the best model fit, consistent with existing literature [24].

No dengue outbreaks were recorded after 2020, coinciding with the implementation of COVID-19–related travel restrictions and the first year following the deployment of the *Wolbachia* program in Nouméa. Consequently, the analysis was restricted to the 2017–2020 period.

A negative association was observed between dengue incidence and the MEI (Table 2). However, the available data did not provide sufficient spatial resolution to formally model the impact of the *Wolbachia* intervention at the archipelago level. Therefore, this variable was included for descriptive purposes only (Supplementary Figure S4).

#### 3.2.3. Hepatitis A Virus (HAV)

Figure 1C illustrates the spatial distribution of the average incidence of hepatitis A. During the 2018 epidemic, cases were predominantly concentrated on the island of Maré.

Time-series analysis of indexed hepatitis A cases, combined with mean precipitation data across the territory, demonstrated a significant association when modelled using a GAM (correlation coefficient: 0.52; Figure 2C).

Consistently, the final model results (Table 2) suggest that the risk of HAV transmission may be influenced by large-scale oceanic variability, as reflected by the MEI.

#### 3.2.4. Leptospirosis

The spatial distribution of leptospirosis cases was predominantly concentrated along the eastern coast and in the northern part of the archipelago, with very few cases reported in the Loyalty Islands Province (Lifou, Ouvéa, and Maré, located east of the main island), Belep (Northern Province), and the Île des Pins (Southern Province). Cases reported in these islands were likely linked to exposure on the main island (Figure 1D). These islands were therefore excluded from the final model. Temporally, most cases occurred between 2020 and 2023 (Figure 2D).

Multivariate analysis identified several socio-economic factors significantly associated with leptospirosis incidence. A cumulative effect of precipitation was observed, with the strongest association detected 28 to 42 days after rainfall peaks (see Supplementary Materials). Specifically, a higher proportion of the population with lower educational attainment (defined as no diploma or only a low level of completion within the school system) was associated with increased leptospirosis incidence (Table 2). In contrast, greater household access to potable water was associated with a lower number of cases, underscoring a clear socio-economic gradient in disease risk.

## 4. Discussion

The unique geographical and socio-demographic context of New Caledonia, combined with the COVID-19 pandemic and the ENSO climatic event, provided a quasi-experimental setting in which to investigate infectious disease dynamics. The archipelago-wide lockdown, implemented within a Zero-COVID strategy, coincided with a prolonged La Niña episode characterized by unusually heavy rainfall.

A key strength of this study lies in the simultaneous analysis of four pathogens with distinct transmission pathways. This approach offers a counterfactual framework to disentangle the respective contributions of climate variability, mobility restrictions, and social determinants in the context of ongoing global health transitions.

Our findings indicate that environmentally transmitted diseases, such as leptospirosis, were significantly associated with precipitation during ENSO-related climatic events, independently of international travel restrictions, but were modulated by socio-economic factors including educational attainment and access to potable water. These results underscore the value of geographic data science in assessing healthcare systems and understanding disease vulnerability patterns [25].

In contrast, airborne and vector-borne diseases—such as influenza and dengue—declined during the lockdown period; however, only influenza resurged following the reopening of borders [26,27,28]. This differential response highlights the complex interplay between environmental drivers, population immunity, mobility, and pathogen-specific transmission dynamics. Meanwhile, hepatitis A, a waterborne infection, declined in parallel with the attenuation of the La Niña event.

Our study revealed several key observations.

**Influenza.** Influenza epidemics disappeared during border closure and re-emerged with a steeper epidemic curve following reopening. This pattern is consistent with observations from other island settings, such as Australia and New Zealand, where influenza activity increased markedly after the lifting of travel restrictions [27,29]. Spatial analyses indicated that influenza incidence was associated with precipitation prior to lockdown, but not thereafter. This temporal shift underscores the impact of mobility restrictions and population-level immunity, in line with global observations. A major contribution of this study is the identification of a decoupling between influenza transmission and ENSO-related meteorological variability (specifically, La Niña conditions) during and after territorial isolation.

**Dengue.** Dengue incidence declined following border closure, and no epidemic was recorded during the first years thereafter. The implementation of the *Wolbachia* program in July 2019 may also have influenced transmission dynamics, in combination with residual population immunity from prior viral circulation [21]. The geographic isolation of the archipelago may have enhanced the effectiveness of the *Wolbachia* intervention, particularly in the capital region of Nouméa and surrounding areas [26,30,31]. However, the temporal overlap between *Wolbachia* deployment and border closure, together with the low number of cases, precluded formal modelling of its independent effect. Disruptions in dengue transmission during the COVID-19 pandemic have also been reported in Southeast Asia [32]. At the time of writing (2025), no further epidemic had been reported in New Caledonia while Fiji, Tonga, and French Polynesia experienced dengue outbreaks (https://www.spc.int/phd/epidemics/ (accessed on 7 May 2025)) contrasting with historical data [30,33]. However, dengue circulation was reported again for the first time in early 2026, at the time of the submission of this paper. The outbreak was mainly observed outside the zones where the *Wolbachia* program was implemented, according to local authorities (La dengue|Direction des Affaires Sanitaires et Sociales de Nouvelle-Calédonie (5 April 2026)).

**Hepatitis A.** Hepatitis A incidence increased during the period of border closure and subsequently declined, describing an epidemic pattern aligned with large-scale oceanic variability and precipitation trends. This trajectory contrasts with reports from other continental and island settings, where hepatitis A incidence decreased during the COVID-19 pandemic [34,35]. Hepatitis A is strongly associated with inadequate sanitation conditions. To date, only one previous study conducted in New Caledonia has described epidemic patterns consistent with our findings [36]. These results highlight the need for further epidemiological research in isolated island settings, particularly in light of the very high seroprevalence (up to 95%) reported in regions such as Micronesia and the Marshall Islands, where distinct endemicity patterns have been observed [37]. The drivers of endemicity in low-lying island contexts remain poorly understood.

**Leptospirosis.** Leptospirosis cases were predominantly concentrated along the eastern coast and in the Northern Province, in correlation with heavy rainfall during La Niña episodes [16]. However, unlike rainfall, the MEI was not consistently associated with leptospirosis incidence across all models [16,38,39]. A one-month lag between rainfall peaks and case occurrence provided the best predictive performance, consistent with recent literature [39,40]. Socio-economic inequalities—particularly limited access to potable water and lower educational attainment—were significantly associated with disease distribution. These findings highlight both structural barriers to healthcare access and the need for culturally appropriate prevention programs.

More broadly, environmental exposure emerged as a central determinant. Infectious diseases linked to water exposure and contact with contaminated soil, such as hepatitis A and leptospirosis, were more sensitive to climatic conditions, independently of travel-related introductions. Higher incidence rates were observed in areas characterized by lower socio-economic status, as reflected by limited access to water, electricity, or transport [1]. These included the eastern coast and Northern Province for leptospirosis and the Loyalty Islands Province for hepatitis A.

Future climate projections suggest a potential intensification of ENSO events, which may amplify the risks posed by emerging and re-emerging infectious diseases. Climate change is therefore likely to exacerbate environmental determinants of transmission. Nevertheless, we acknowledge that the study period was relatively short for establishing robust long-term climatic trends.

Social disparities were examined through multiple indicators. Influenza appeared to be largely independent of the socio-economic variables considered, whereas other diseases displayed marked social gradients. Civil unrest in New Caledonia in May 2024, triggered by social inequalities, ethnic divisions, and colonial legacies, further underscores the urgency of addressing structural determinants of health. Education, mobility, and access to safe water act as proxies for these disparities and reinforce the need for community-centered, equity-oriented health strategies [1,41].

Although clinical outcomes and immunization histories were not assessed, this study provides insights into infectious disease circulation before, during, and after territorial isolation. Our analysis suggests that dengue dynamics likely reflected a combined effect of acquired immunity, biocontrol interventions, and border closure [26]. In contrast, the two-year interruption of influenza circulation was followed by an atypically intense resurgence, independent of meteorological factors, highlighting the importance of timely vaccination strategies and the kinetics of waning immunity.

This study has limitations inherent to retrospective hospital database analyses. The use of postal codes as proxies for infection location may have introduced spatial misclassification, and improvements in diagnostic capacity over time may have influenced case detection. Nonetheless, consistent case definitions were applied for each disease, supporting the robustness of our findings. Meteorological indicators may not fully capture local microclimatic variability. Further analyses incorporating additional parameters, such as temperature, and network-based approaches to characterize human mobility and environmental transmission pathways, would strengthen future research.

## 5. Conclusions

The lockdown implemented in New Caledonia, coinciding with the prolonged 2020–2023 La Niña event [42], provided a unique opportunity to examine infectious disease dynamics independently of travel-related introductions. Environmentally transmitted and waterborne diseases increased during periods of higher precipitation associated with La Niña conditions and variations in the MEI, whereas influenza resurged following border reopening, underscoring the impact of island isolation on population immunity. Community-level determinants and access to healthcare remain critical factors in mitigating climate-sensitive disease risks. Targeted, locally driven interventions are therefore essential to strengthen culturally informed, equitable, and climate-resilient health strategies.

## Figures and Tables

**Table 1:** Cases and annual incidences of influenza (biologically confirmed), dengue, Hepatitis A virus and leptospirosis during the three time periods: before, during and after the border closure (reported in days of duration and by yearly means in the incidence calculation).

Period	Influenza (Biologically Confirmed)		Dengue		Viral hepatitis A		Leptospirosis	
Border Status Period	Number of Cases	Incidence per 100,000 Inhabitants/Year	Number of Cases	Incidence per 100,000 Inhabitants/Year	Number of Cases	Incidence per 100,000 Inhabitants/Year	Number of Cases	Incidence per 100,000 Inhabitants/Year
**Before (1176 days)**	1072	123	7213	829	14	2	242	28
**Closed (649 days)**	0	0	153	32	144	30	256	53
**After (729 days)**	950	176	11	2	7	2	380	71
**Total (over 7 years)**	2022	107	7377	390	165	9	878	46

**Table 2:** Fixed effects of GAMM for Influenza, Dengue, Hepatitis A virus (HAV), and Leptospirosis epidemics using communal district as random effect.

Variable	Coefficient	Standard Error	Odds Ratio (95% CI)	*p*-Value
*Influenza before border closure*				
Mean daily precipitation over each commune (mm/d)	−0.4	0.1	0.7 (0.6–0.8)	<0.001
*Influenza after border closure*				
Mean daily precipitation over each commune (mm/d)	0.2	0.2	1.2 (0.8–1.8)	0.301
*Dengue before border closure*				
MEI	−0.3	0.1	0.78 (0.6–1)	0.049
*Hepatitis A*				
MEI	0.6	0.3	1.8 (1–3.3)	0.042
Percentage of inhabitants with a BEPC or lower	−0.1	0.0	0.9 (0.9–1)	0.052
*Leptospirosis*				
Mean daily precipitation over each commune (mm/d) averaged from weekly precipitation				
Precipitation week 1	−0.04	0.05	0.96 (0.9–1.1)	0.46
Precipitation week 2	0.11	0.05	1.12 (1–1.23)	0.017
Precipitation week 3	−0.11	0.05	0.90 (0.8–1)	0.025
Precipitation week 4	0.05	0.02	1.05 (1–1.1)	0.037
Precipitation week 5	0.11	0.02	1.12 (1.1–1.2)	<0.001
Percentage of inhabitants who walks as their main mode of transport	0.3	0.2	1.33 (1–1.9)	0.08
Percentage of inhabitants with water supply	−0.4	0.1	0.7 (0.5–0.9)	0.015
Percentage of inhabitants with a BEPC or lower	0.04	0.01	1.04 (1.02–1.1)	0.008

Note: BEPC: first grade diploma obtained at 14–15 years old. Odds ratios were calculated based on the exponent of the coefficient. CI: Confidence Interval at 95%; MEI: Multivariate El Niño/Southern Oscillation (ENSO) index.

## Data Availability

The data presented in this study are available on request from the corresponding author. We are prepared to share our data according to French and New Caledonian laws on health data upon specific request to PHM (phmoury@chu-grenoble.fr).

## Supplementary Materials

The following supporting information can be downloaded at: https://www.mdpi.com/article/10.3390/epidemiologia7030070/s1, Figure S1: Meteorological conditions in New-Caledonia; Figure S2: Correlations between socio-economic variables–Principal Component Analysis (PCA); Figure S3: Evolution of cases and moving 7 days averages of influenza, hepatitis A virus HAV, dengue and leptospirosis with their predicted models (orange) over the New-Caledonia; Figure S4: Dengue incidence per habitant and Wolbachia; Figure S5: Cumulative effect of precipiration on leptospirosis using a GAMM method and a distributed lag non-linear model (DLNM) embedded; Figure S6: Humidity and rain standardised values correlation represented through time; Figure S7: Temperature and rain standardised values correlation represented through time; Table S1: GAMM complete model for Influenza, Dengue, Hepatitis A virus (HAV), and leptospirosis with the complete variables of the formula MEI index and health care facilities. The refences [43,44,45,46,47,48,49,50,51,52] are cited in the Supplementary Materials.

## Abbreviations

The following abbreviations are used in this manuscript:

CHT	Centre Hospitalier Territorial Gaston-Bourret de Nouvelle-Calédonie
CI	Confidence Interval
ENSO	El Niño/Southern Oscillation (ENSO)
GAM	Generalized Additive Model
GAMM	Generalized Additive Mixed Model
HAV	Hepatitis A virus
MEI	bi-monthly Multivariate El Niño/Southern Oscillation (ENSO) index
OR	Odds Ratio
PCA	Principal component analysis
PICTS	Pacific Island Countries and Territories

## References

[1] Moury P.-H., Tromhae M., Cazorla C., Série M., Flahault A., Couadau E., Fleury C., Mangeas M., De Greslan T. (2025). Colonial Transition as a Major Mediator of Global Health Transition: Lessons from the 2024 New Caledonia Crisis. J. Glob. Health.

[2] Shanks G.D., Wilson N., Kippen R., Brundage J.F. (2018). The Unusually Diverse Mortality Patterns in the Pacific Region during the 1918–1921 Influenza Pandemic: Reflections at the Pandemic’s Centenary. Lancet Infect. Dis..

[3] Sand C. (2023). Hécatombe océanienne. Histoire de la dépopulation du Pacifique et ses conséquences (XVIe-XXe siècle).

[4] Kerbaj J., Cazorla C., De Greslan T., Serie M., Gourinat A.-C., Marot B. (2020). COVID-19: The New Caledonia Experience. Clin. Infect. Dis..

[5] Moury P.-H., Ochida N., Motiejunaite J., Collart V., Série M., Gervolino S., Mangeas M., Bouvier J.-B., Couadau E., Mebazaa A. (2023). Impact of Lockdown on Cardiovascular Disease Hospitalizations in a Zero-COVID-19 Country. Public Health.

[6] Moury P.-H., Gourinat A.-C., Riou O., Laumond S., Dupont-Rouzeyrol M., Cazorla C., Mangeas M. (2021). Successful COVID-19 Elimination after an Alpha Variant Outbreak in a “Safe Travel Zone”. Travel Med. Infect. Dis..

[7] Ochida N., Dupont-Rouzeyrol M., Moury P.-H., Demaneuf T., Gourinat A.-C., Mabon S., Jouan M., Cauchemez S., Mangeas M. (2023). Evaluating the Strategies to Control SARS-CoV-2 Delta Variant Spread in New Caledonia, a Zero-COVID Country until September 2021. IJID Reg..

[8] Murphy B.F., Power S.B., McGree S. (2014). The Varied Impacts of El Niño–Southern Oscillation on Pacific Island Climates. J. Clim..

[9] Moron V., Barbero R., Robertson A.W. (2016). Subseasonal-to-Interannual Variability of Rainfall over New Caledonia (SW Pacific). Clim. Dyn..

[10] Inizan C., Tarantola A., O’Connor O., Mangeas M., Pocquet N., Forfait C., Descloux E., Gourinat A.-C., Pfannstiel A., Klement-Frutos E. (2019). Dengue in New Caledonia: Knowledge and Gaps. Trop. Med. Infect. Dis..

[11] Ochida N., Mangeas M., Dupont-Rouzeyrol M., Dutheil C., Forfait C., Peltier A., Descloux E., Menkes C. (2022). Modeling Present and Future Climate Risk of Dengue Outbreak, a Case Study in New Caledonia. Environ. Health.

[12] Togami E., Chiew M., Lowbridge C., Biaukula V., Bell L., Yajima A., Eshofonie A., Saulo D., Hien D.T.H., Otsu S. (2023). Epidemiology of Dengue Reported in the World Health Organization’s Western Pacific Region, 2013–2019. West. Pac. Surveill. Response J..

[13] (2012). Western Pacific Region Global Influenza Surveillance and Response System Epidemiological and Virological Characteristics of Influenza in the Western Pacific Region of the World Health Organization, 2006–2010. PLoS ONE.

[14] Koff R.S. (1998). Hepatitis A. Lancet.

[15] Goarant C., Laumond-Barny S., Perez J., Vernel-Pauillac F., Chanteau S., Guigon A. (2009). Outbreak of Leptospirosis in New Caledonia: Diagnosis Issues and Burden of Disease. Trop. Med. Int. Health.

[16] Douchet L., Menkes C., Herbreteau V., Larrieu J., Bador M., Goarant C., Mangeas M. (2024). Climate-Driven Models of Leptospirosis Dynamics in Tropical Islands from Three Oceanic Basins. PLoS Neglected Trop. Dis..

[17] Findlater A., Bogoch I.I. (2018). Human Mobility and the Global Spread of Infectious Diseases: A Focus on Air Travel. Trends Parasitol..

[18] Mora C., McKenzie T., Gaw I.M., Dean J.M., von Hammerstein H., Knudson T.A., Setter R.O., Smith C.Z., Webster K.M., Patz J.A. (2022). Over Half of Known Human Pathogenic Diseases Can Be Aggravated by Climate Change. Nat. Clim. Chang..

[19] Barbero R., Moron V. (2011). Seasonal to Decadal Modulation of the Impact of El Niño–Southern Oscillation on New Caledonia (SW Pacific) Rainfall (1950–2010). J. Geophys. Res. Atmos..

[20] Zhang T., Hoell A., Perlwitz J., Eischeid J., Murray D., Hoerling M., Hamill T.M. (2019). Towards Probabilistic Multivariate ENSO Monitoring. Geophys. Res. Lett..

[21] Utarini A., Indriani C., Ahmad R.A., Tantowijoyo W., Arguni E., Ansari M.R., Supriyati E., Wardana D.S., Meitika Y., Ernesia I. (2021). Efficacy of Wolbachia-Infected Mosquito Deployments for the Control of Dengue. New Engl. J. Med..

[22] Kurz C.F. (2017). Tweedie Distributions for Fitting Semicontinuous Health Care Utilization Cost Data. BMC Med. Res. Methodol..

[23] Ma R., Yan G., Hasan M.T. (2018). Tweedie Family of Generalized Linear Models with Distribution-Free Random Effects for Skewed Longitudinal Data. Stat. Med..

[24] Fletcher C., Moirano G., Alcayna T., Rollock L., Van Meerbeeck C.J., Mahon R., Trotman A., Boodram L.-L., Browne T., Best S. (2025). Compound and Cascading Effects of Climatic Extremes on Dengue Outbreak Risk in the Caribbean: An Impact-Based Modelling Framework with Long-Lag and Short-Lag Interactions. Lancet Planet. Health.

[25] Weinberger D., Baroux N., Grangeon J.-P., Ko A.I., Goarant C. (2014). El Niño Southern Oscillation and Leptospirosis Outbreaks in New Caledonia. PLoS Neglected Trop. Dis..

[26] Li N., Feng Y., Vrancken B., Chen Y., Dong L., Yang Q., Kraemer M.U.G., Pybus O.G., Zhang H., Brady O.J. (2021). Assessing the Impact of COVID-19 Border Restrictions on Dengue Transmission in Yunnan Province, China: An Observational Epidemiological and Phylogenetic Analysis. Lancet Reg. Health West. Pac..

[27] O’Neill G.K., Taylor J., Kok J., Dwyer D.E., Dilcher M., Hua H., Levy A., Smith D., Minney-Smith C.A., Wood T. (2023). Circulation of Influenza and Other Respiratory Viruses during the COVID-19 Pandemic in Australia and New Zealand, 2020–2021. West. Pac. Surveill. Response J..

[28] Taylor-Salmon E., Hill V., Paul L.M., Koch R.T., Breban M.I., Chaguza C., Sodeinde A., Warren J.L., Bunch S., Cano N. (2024). Travel Surveillance Uncovers Dengue Virus Dynamics and Introductions in the Caribbean. Nat. Commun..

[29] Huang Q.S., Turner N., Wood T., Anglemyer A., McIntyre P., Aminisani N., Dowell T., Trenholme A., Byrnes C., Balm M. (2024). Impact of the COVID-19 Related Border Restrictions on Influenza and Other Common Respiratory Viral Infections in New Zealand. Influenza Other Respir. Viruses.

[30] Zellweger R.M., Cano J., Mangeas M., Taglioni F., Mercier A., Despinoy M., Menkès C.E., Dupont-Rouzeyrol M., Nikolay B., Teurlai M. (2017). Socioeconomic and Environmental Determinants of Dengue Transmission in an Urban Setting: An Ecological Study in Nouméa, New Caledonia. PLoS Negl. Trop. Dis..

[31] Teurlai M., Menkès C.E., Cavarero V., Degallier N., Descloux E., Grangeon J.-P., Guillaumot L., Libourel T., Lucio P.S., Mathieu-Daudé F. (2015). Socio-Economic and Climate Factors Associated with Dengue Fever Spatial Heterogeneity: A Worked Example in New Caledonia. PLoS Negl. Trop. Dis..

[32] Chen Y., Li N., Lourenço J., Wang L., Cazelles B., Dong L., Li B., Liu Y., Jit M., Bosse N.I. (2022). Measuring the Effects of COVID-19-Related Disruption on Dengue Transmission in Southeast Asia and Latin America: A Statistical Modelling Study. Lancet Infect. Dis..

[33] Inizan C., Minier M., Prot M., O’Connor O., Forfait C., Laumond S., Marois I., Biron A., Gourinat A.-C., Goujart M.-A. (2021). Viral Evolution Sustains a Dengue Outbreak of Enhanced Severity. Emerg. Microbes Infect..

[34] Rzymski P., Zarębska-Michaluk D., Genowska A., Tyszko P., Strukcinskiene B., Flisiak R. (2024). Trends of Hepatitis A Virus Infection in Poland: Assessing the Potential Impact of the COVID-19 Pandemic and War in Ukraine. Viruses.

[35] Murakoshi K., Mori H., Prasertbun R., Valenti S., Krokva D., Remez D., Mahittikorn A., Hadano Y., Naito T. (2025). Hepatitis A Epidemics in Japan, France, and Thailand from 2007 to 2021, Highlighting a Post-COVID-19 Decline. Sci. Rep..

[36] Berlioz-Arthaud A., Barny S., Yvon J.F., Roque-Afonso A.M., Dussaix E. (2008). Laboratory based hepatitis A surveillance in New Caledonia: From an endemic to an epidemic pattern (1986–2007). Bull. Soc. Pathol. Exot..

[37] Fischer G.E., Thompson N., Chaves S.S., Bower W., Goldstein S., Armstrong G., Williams I., Bialek S. (2009). The Epidemiology of Hepatitis A Virus Infections in Four Pacific Island Nations, 1995–2008. Trans. R. Soc. Trop. Med. Hyg..

[38] Bierque E., Thibeaux R., Girault D., Soupé-Gilbert M.-E., Goarant C. (2020). A Systematic Review of Leptospira in Water and Soil Environments. PLoS ONE.

[39] Thibeaux R., Genthon P., Govan R., Selmaoui-Folcher N., Tramier C., Kainiu M., Soupé-Gilbert M.-E., Wijesuriya K., Goarant C. (2024). Rainfall-Driven Resuspension of Pathogenic Leptospira in a Leptospirosis Hotspot. Sci. Total Environ..

[40] Govan R., Scherrer R., Fougeron B., Laporte-Magoni C., Thibeaux R., Genthon P., Fournier-Viger P., Goarant C., Selmaoui-Folcher N. (2025). Spatio-Temporal Risk Prediction of Leptospirosis: A Machine-Learning-Based Approach. PLoS Negl. Trop. Dis..

[41] Baroux N., Maire L., Cadic L., Lemaitre A.-F., Borceux P., Glasman B. (2024). Riots in New Caledonia: Impact of Constrained Management on Peritoneal Dialysis Patients. Bull. Dial. Domic..

[42] Geng T., Jia F., Cai W., Wu L., Gan B., Jing Z., Li S., McPhaden M.J. (2023). Increased Occurrences of Consecutive La Niña Events under Global Warming. Nature.

[43] Tubiana S., Mikulski M., Becam J., Lacassin F., Lefèvre P., Gourinat A.-C., Goarant C., D’Ortenzio E. (2013). Risk Factors and Predictors of Severe Leptospirosis in New Caledonia. PLoS Negl. Trop. Dis..

[44] Pocquet N., O’Connor O., Flores H.A., Tutagata J., Pol M., Hooker D.J., Inizan C., Russet S., Duyvestyn J.M., Pacidônio E.C. (2021). Assessment of Fitness and Vector Competence of a New Caledonia wMel Aedes Aegypti Strain before Field-Release. PLoS Negl. Trop. Dis..

[45] Guibreteau H., Tarantola A., Goarant C., Gervolino S., Gourinat A.-C., Colot J., Cazorla C., Klement-Frutos E. (2022). Clinical Evaluation of the Modified Faine Criteria in Patients Admitted with Suspected Leptospirosis to the Territorial Hospital, New Caledonia, 2018 to 2019. Am. J. Trop. Med. Hyg..

[46] Severi E., Tavoschi L., Carrillo-Santisteve P., Westrell T., Marrone G., Giesecke J., Lopalco P. (2023). Hepatitis A Notifications in the EU/EEA, 2010-2019: What Can We Learn from Case Reporting to the European Surveillance System?. Eurosurveillance.

[47] Pongpan S., Thanatrakolsri P., Vittaporn S., Khamnuan P., Daraswang P. (2023). Prognostic Factors for Leptospirosis Infection Severity. Trop. Med. Infect. Dis..

[48] Wu X., Lang L., Ma W., Song T., Kang M., He J., Zhang Y., Lu L., Lin H., Ling L. (2018). Non-Linear Effects of Mean Temperature and Relative Humidity on Dengue Incidence in Guangzhou, China. Sci. Total Environ..

[49] Cabrera M., Taylor G. (2019). Modelling Spatio-Temporal Data of Dengue Fever Using Generalized Additive Mixed Models. Spat. Spatio-Temporal Epidemiol..

[50] Ndii M.Z. (2020). Modelling the Use of Vaccine and Wolbachia on Dengue Transmission Dynamics. Trop. Med. Infect. Dis..

[51] Zhu H., Chen S., Lu W., Chen K., Feng Y., Xie Z., Zhang Z., Li L., Ou J., Chen G. (2022). Study on the Influence of Meteorological Factors on Influenza in Different Regions and Predictions Based on an LSTM Algorithm. BMC Public Health.

[52] Chen Z., Liu Y., Yue H., Chen J., Hu X., Zhou L., Liang B., Lin G., Qin P., Feng W. (2023). The Role of Meteorological Factors on Influenza Incidence among Children in Guangzhou China, 2019–2022. Front. Public Health.

