# Supplementary material for: Influence of Both La Nina and Island Isolation During COVID-19 on the Epidemiology of Infectious Diseases in New Caledonia
Source: Epidemiologia (Basel). 2026 May 21;7(3):70. doi: 10.3390/epidemiologia7030070 (PMC13214734; doi:10.3390/epidemiologia7030070)
Supplement: Supplementary file 1 [file epidemiologia-07-00070-s001.zip › epidemiologia-4249715-supplementary.pdf]

Supplementary materials:

1. **Four infectious diseases in New Caledonia context: four models**

Influenza, characterized by seasonal epidemics and pandemics, has had dramatic consequences in the Pacific area historically and is a significant burden on native populations [1,2]. New Caledonia classically experiences two seasonal peaks: one during winter (due to southern strains) and another during summer (due to northern strains) [3].

Dengue is an emerging and re-emerging mosquito-borne disease transmitted in New Caledonia by *Aedes aegypti*. Dengue incidence increased in Asia and Western Pacific Region in the last 20 years [4]. New Caledonia experienced three major dengue outbreaks in the decade starting in 2010,[5] and modelling data suggest an increased risk of yearly epidemics towards 2100 in a context of climate change [6]. As a consequence, the *Wolbachia* bio-control strategy program was implemented in New Caledonia in 2019 [7,8].

Hepatitis A, a viral food-borne, water-borne, and travel-related disease, can cause death during pregnancy or childhood. Monitoring its epidemic features is crucial to prevent extended crises. In New Caledonia, Hepatitis A was never found as endemic but only triggered epidemics [9,10].

Leptospirosis is a bacterial disease caused by a spirochete, potentially severe with reported case fatality rates up to 13%, and includes recurrent epidemics in New Caledonia considered as one of the world hotspot that are strongly influenced by rainfall data [11–14]. Therefore, local research programs have studied the specific conditions that promoted the outbreaks including the ENSO pattern and zoonotic epidemiology [15–17].

2. **COVID-19 Lockdown Measures in New Caledonia: Implementation and Impact**

In 2020, New Caledonia adopted a rigorous "zero-COVID" strategy in response to the emerging global pandemic, implementing its first strict lockdown measures to prevent the introduction and spread of SARS-CoV-2[18]. These early interventions included strict border closures on the 20th oh March, mandatory quarantine for incoming travelers, and restrictions on public gatherings with a one-month period of full inhouse isolation for the inhabitants on the 23rd of March after the detection of the first case at the border. The territory's geographic isolation and early elimination strategy allowed it to remain largely COVID-free during the initial phases of the pandemic[19]. Masks were compulsory in public during two month and gathering prohibited. These measures were supported by community leaders, who emphasized the historical vulnerability of the Kanak population to introduced infectious diseases.

By March 2021, the detection of the Alpha variant necessitated a second lockdown, as community transmission threatened the territory's fragile epidemiological balance[20]. At this time, less than 10% of the population was vaccinated, and public health authorities enforced a strict in-home isolation during a one-month period followed by a nightly curfew, mandatory mask-wearing in all indoor and public spaces, and limits on social gatherings.

The most severe COVID-19 introduction occurred in September 2021, with the highly transmissible Delta variant. This outbreak overwhelmed the local health system, resulting in nearly 100 deaths per 100,000 population within three months, despite less than 30% vaccination coverage. The response included a territory-wide curfew, further restrictions on movement, and the closure of non-essential services. Travel restrictions were tightened, with mandatory testing for unvaccinated travellers and the closure of airports in the Loyalty Islands.[21,22] Masks were compulsory in public during two month and gathering prohibited then restricted during November 2021.

The lockdown measures were gradually eased by December 2021, with the lifting of the curfew and relaxation of mask mandates, although some restrictions on large gatherings remained in place until early 2022. Overall, New-Caledonia experienced periods COVID-19-free without restrictions during theses periods. No social distancing was in place and travel in the archipelago was not reduced. In terms of laboratory testing, no limitation was assessed due to the absence of clinical overload that was studied in the setting of cardiopathy acute illnesses[19]. Given the patterns and its sides in terms of cases distribution and figures, we may reasonably think in this paper that we assessed true epidemic shifts rather than artefacts of pandemic periods.

3. **Data collections: *Wolbachia***

The *Wolbachia* program was included in the analysis for representation and descriptive purposes (see supplementary materials). In this study, we used a linear progressive efficacy of *Wolbachia* on cases detection obtained by the end of the year 2021 at 77% in the communal district of Noumea, and by the end of year 2023 in Dumbea and Mont-Dore [7]. The effect of *Wolbachia* was assumed to be exponentiate to fit the progressive introduction, non-linear and instability of the model [23–25].

4. **Meteorological analysis**

Rainfall and temperature were higher after the first lockdown the 20th of March 2020 whether it was measured or calculated from satellites measurements without significant differences when considering the whole territory (supplementary figure 1, panel A and B). We concluded that no corrections were needed when using the satellite teledetection. The ENSO index MEI showed a pattern towards La-Ninã phenomenon during the Island lockdown (Supplementary figure 1 panel C). The MEI is anticorrelated with precipitation at -0.5 (p=0.05) over New Caledonia as, with MEI negative phases indicating La Niña states (enhanced rain) and positive MEI indicating El Niño (weakened rainfall).

To investigate the temporal relationship between rainfall (RR) and humidity (UN) or maximal temperature (TX), both variables were first aggregated by date at a monthly time step to ensure temporal alignment. Given their different physical units (millimeters for precipitation and percentage for humidity), a standardization step was applied using z-score normalization:

- RR_scaled = standardized rainfall
- UN_scaled = standardized humidity
- TX_scaled = standardized maximal temperature

This transformation allows both variables to be compared on the same scale without altering their temporal variability. A smoothing step was then applied to both time series (RR_smooth and UN_smooth) using a locally weighted regression (LOESS) to reduce short-term noise and highlight underlying temporal trends. Finally, both smoothed and standardized series were plotted over time to visually assess co-variability and potential synchronicity between rainfall events and atmospheric humidity dynamics. The Pearson correlation coefficient between smoothed rain, humidity and heat were computed to quantify the strength of their linear relationship.

The time series plot shows a strong temporal co-variation between rainfall (RR) and humidity (UN), with both variables exhibiting synchronized peaks over the study period.

The Pearson correlation coefficient between smoothed rainfall and humidity was: r = 0.84 (**supplementary figure 6)**, and r = 0.69 for temperature (**supplementary figure 7**). These results indicated a strong positive relationship, suggesting that increases in land humidity or temperature were closely associated with increases in rain fall.

5. Statistical analysis

Spleen effect

In the GAMM framework, the spline effect for time is used to flexibly model non-linear temporal trends in the outcome that are not adequately captured by a simple linear or parametric function. Rather than assuming a fixed shape (e.g., linear or quadratic), splines allow the relationship between time and the response variable to be data-driven and smooth, adapting to gradual increases, decreases, or cyclical patterns over the study period.

Specifically, the spline represents time as a series of piecewise polynomial functions joined at defined points: the knots. Also, there are continuity constraints that ensure smooth transitions between segments. This approach enables the model to account for long-term trends, seasonality, and unmeasured temporal confounding, while avoiding overfitting through penalization of excessive curves.

Including time as a spline therefore improves model fit and inference by capturing underlying temporal structure. It ensured that estimated associations with precipitation and MEI are not biased by residual time-dependent patterns in the data.

Lag determination

Influenza

Due to the short incubation time around 2 days and the disparities in the social access to health care, we did use a time lag for influenza [26,27]. Further, we used spline technic to account for time variations.

Dengue

We fitted GAMM with a Tweedie distribution ($p = 1.5$, log link) to estimate the association between rainfall and incidences. Lagged precipitation variables were created for delays from 0 to 70 days (step = 7 days), and models were compared using AIC to identify the lag providing the best fit. Temporal trends were captured using a cubic spline, with commune-level random effects and socio-environmental covariates.

Hepatitis A

We did not use lag period for hepatitis A due to the correlation with the MEI which represents longer trends. Further, we used spline technics to account for time variations (short or long term).

Leptospirosis

For leptospirosis, to assess the delayed effects of precipitation on case incidence, we fitted a distributed lag non-linear model (DLNM) embedded in a generalized additive mixed model (GAM) with a Tweedie ($p = 1.5$) distribution. The response variable was the population-standardized case ratio. The exposure was the weekly mean precipitation, modelled with a cross-basis function allowing for non-linear effects across lags of up to 70 days. Temporal trends were controlled with a cubic spline on date, and commune-level heterogeneity was modelled using a random effect. The DLNM GAM indicated a significant cumulative effect of precipitation at intermediate lags. For example, the 3rd and 4th basis functions of the cross-basis were statistically significant ($p = 0.015$ and $p = 0.006$, respectively), suggesting a delayed response of case incidence approximately 28–42 days after rainfall peaks. Model selection and goodness-of-fit were evaluated using AIC, significance of parametric coefficients, and approximate significance of smooth terms. Compared with the base GAM including only the current precipitation, the DLNM GAM substantially improved model fit, indicating that rainfall affects incidence with a delayed and cumulative effect over several weeks (see supplementary figure 5)

6. References

[1] G.D. Shanks, N. Wilson, R. Kippen, J.F. Brundage, The unusually diverse mortality patterns in the Pacific region during the 1918–21 influenza pandemic: reflections at the pandemic's centenary, The Lancet Infectious Diseases 18 (2018) e323–e332. https://doi.org/10.1016/S1473-3099(18)30178-6.

[2] C. Sand, Hécatombe océanienne. Histoire de la dépopulation du Pacifique et ses conséquences (XVIe-XXe siècle), Au vent des îles, Papeete, n.d. https://auventdesiles.pf/catalogue/collections/sciences-humaines/hecatombe-oceanienne-histoire-de-la-depopulation-du-pacifique-et-ses-consequences-xvie-xxe-siecle/ (accessed September 6, 2024).

[3] Western Pacific Region Global Influenza Surveillance and Response System, Epidemiological and virological characteristics of influenza in the Western Pacific Region of the World Health Organization, 2006-2010, PLoS One 7 (2012) e37568. https://doi.org/10.1371/journal.pone.0037568.

[4] E. Togami, M. Chiew, C. Lowbridge, V. Biaukula, L. Bell, A. Yajima, A. Eshofonie, D. Saulo, D.T.H. Hien, S. Otsu, T.C. Dai, M.S. Ngon, C.-K. Lee, R. Tsuyuoka, L. Tuseo, A. Khalakdina, V. Kab, R.R. Abeyasinghe, R.P. Yadav, P. Esguerra, S. Casey, C.P. Soo, M. Fukusumi, T. Matsui, B. Olowokure, Epidemiology of dengue reported in the World Health Organization's Western Pacific Region, 2013–2019, Western Pac Surveill Response J 14 (2023) 1–16. https://doi.org/10.5365/wpsar.2023.14.1.973.

[5] C. Inizan, A. Tarantola, O. O'Connor, M. Mangeas, N. Pocquet, C. Forfait, E. Descloux, A.-C. Gourinat, A. Pfannstiel, E. Klement-Frutos, C. Menkes, M. Dupont-Rouzeyrol, Dengue in New Caledonia: Knowledge and Gaps, Tropical Medicine and Infectious Disease 4 (2019) 95. https://doi.org/10.3390/tropicalmed4020095.

[6] N. Ochida, M. Mangeas, M. Dupont-Rouzeyrol, C. Dutheil, C. Forfait, A. Peltier, E. Descloux, C. Menkes, Modeling present and future climate risk of dengue outbreak, a case study in New Caledonia, Environ Health 21 (2022) 20. https://doi.org/10.1186/s12940-022-00829-z.

[7] A. Utarini, C. Indriani, R.A. Ahmad, W. Tantowijoyo, E. Arguni, M.R. Ansari, E. Supriyati, D.S. Wardana, Y. Meitika, I. Ernesia, I. Nurhayati, E. Prabowo, B. Andari, B.R. Green, L. Hodgson, Z. Cutcher, E. Rancès, P.A. Ryan, S.L. O'Neill, S.M. Dufault, S.K. Tanamas, N.P. Jewell, K.L. Anders, C.P. Simmons, Efficacy of Wolbachia-Infected Mosquito Deployments for the Control of Dengue, New England Journal of Medicine 384 (2021) 2177–2186. https://doi.org/10.1056/NEJMoa2030243.

[8] N. Pocquet, O. O'Connor, H.A. Flores, J. Tutagata, M. Pol, D.J. Hooker, C. Inizan, S. Russet, J.M. Duyvestyn, E.C. Pacidônio, D. Girault, D. da Silva Gonçalves, M. Minier, F. Touzain, E. Chalus, K. Lucien, F. Cheilan, T. Derycke, S. Laumond, C.P. Simmons, M. Dupont-Rouzeyrol, N. Rossi, Assessment of fitness and vector competence of a New Caledonia wMel Aedes aegypti strain before field-release, PLoS Negl Trop Dis 15 (2021) e0009752. https://doi.org/10.1371/journal.pntd.0009752.

[9] R.S. Koff, Hepatitis A, The Lancet 351 (1998) 1643–1649. https://doi.org/10.1016/S0140-6736(98)01304-X.

[10] E. Severi, L. Tavoschi, P. Carrillo-Santisteve, T. Westrell, G. Marrone, J. Giesecke, P. Lopalco, Hepatitis A notifications in the EU/EEA, 2010-2019: what can we learn from case reporting to the European Surveillance System?, Euro Surveill 28 (2023) 2200575. https://doi.org/10.2807/1560-7917.ES.2023.28.19.2200575.

[11] H. Guibreteau, A. Tarantola, C. Goarant, S. Gervolino, A.-C. Gourinat, J. Colot, C. Cazorla, E. Klement-Frutos, Clinical Evaluation of the Modified Faine Criteria in Patients Admitted with Suspected Leptospirosis to the Territorial Hospital, New Caledonia, 2018 to 2019, Am J Trop Med Hyg 106 (2022) 486–493. https://doi.org/10.4269/ajtmh.21-0352.

[12] S. Tubiana, M. Mikulski, J. Becam, F. Lacassin, P. Lefèvre, A.-C. Gourinat, C. Goarant, E. D'Ortenzio, Risk factors and predictors of severe leptospirosis in New Caledonia, PLoS Negl Trop Dis 7 (2013) e1991. https://doi.org/10.1371/journal.pntd.0001991.

[13] S. Pongpan, P. Thanatrakolsri, S. Vittaporn, P. Khamnuan, P. Daraswang, Prognostic Factors for Leptospirosis Infection Severity, Trop Med Infect Dis 8 (2023) 112. https://doi.org/10.3390/tropicalmed8020112.

[14] L. Douchet, C. Menkes, V. Herbreteau, J. Larrieu, M. Bador, C. Goarant, M. Mangeas, Climate-driven models of leptospirosis dynamics in tropical islands from three oceanic basins, PLOS Neglected Tropical Diseases 18 (2024) e0011717. https://doi.org/10.1371/journal.pntd.0011717.

[15] C. Goarant, S. Laumond-Barny, J. Perez, F. Vernel-Pauillac, S. Chanteau, A. Guigon, Outbreak of leptospirosis in New Caledonia: diagnosis issues and burden of disease, Trop Med Int Health 14 (2009) 926–929. https://doi.org/10.1111/j.1365-3156.2009.02310.x.

[16] E. Bierque, R. Thibeaux, D. Girault, M.-E. Soupé-Gilbert, C. Goarant, A systematic review of Leptospira in water and soil environments, PLoS One 15 (2020) e0227055. https://doi.org/10.1371/journal.pone.0227055.

[17] D. Weinberger, N. Baroux, J.-P. Grangeon, A.I. Ko, C. Goarant, El Niño Southern Oscillation and Leptospirosis Outbreaks in New Caledonia, PLOS Neglected Tropical Diseases 8 (2014) e2798. https://doi.org/10.1371/journal.pntd.0002798.

[18] J. Kerbaj, C. Cazorla, T. De Greslan, M. Serie, A.-C. Gourinat, B. Marot, COVID-19: The New Caledonia experience, Clin Infect Dis (2020) ciaa600. https://doi.org/10.1093/cid/ciaa600.

[19] P.-H. Moury, N. Ochida, V. Collart, M. Série, S. Gervolino, M. Mangeas, J.-B. Bouvier, E. Couadau, M. Dupont-Rouzeyrol, Impact of Lockdown on Acute Heart Diseases: Lessons from a COVID-19 Free Country, Social Science Research Network, Rochester, NY, 2021. https://doi.org/10.2139/ssrn.3822289.

[20] P.-H. Moury, A.-C. Gourinat, O. Riou, S. Laumond, M. Dupont-Rouzeyrol, C. Cazorla, M. Mangeas, Successful COVID-19 elimination after an alpha variant outbreak in a "safe travel zone," Travel Medicine and Infectious Disease 44 (2021) 102202. https://doi.org/10.1016/j.tmaid.2021.102202.

[21] N. Ochida, M. Dupont-Rouzeyrol, P.-H. Moury, T. Demaneuf, A.-C. Gourinat, S. Mabon, M. Jouan, S. Cauchemez, M. Mangeas, Evaluating the strategies to control SARS-CoV-2 Delta variant spread in New Caledonia, a zero-COVID country until September 2021, IJID Reg 8 (2023) 64–70. https://doi.org/10.1016/j.ijregi.2023.06.004.

[22] P.-H. Moury, M. Tromhae, C. Cazorla, M. Série, A. Flahault, E. Couadau, C. Fleury, M. Mangeas, T. De Greslan, Colonial transition as a major mediator of global health transition: lessons from the 2024 New Caledonia crisis, J Glob Health 15 (2025) 03004. https://doi.org/10.7189/jogh.15.03004.

[23] X. Wu, L. Lang, W. Ma, T. Song, M. Kang, J. He, Y. Zhang, L. Lu, H. Lin, L. Ling, Non-linear effects of mean temperature and relative humidity on dengue incidence in Guangzhou, China, Science of The Total Environment 628–629 (2018) 766–771. https://doi.org/10.1016/j.scitotenv.2018.02.136.
[24] M. Cabrera, G. Taylor, Modelling spatio-temporal data of dengue fever using generalized additive mixed models, Spatial and Spatio-Temporal Epidemiology 28 (2019) 1–13. https://doi.org/10.1016/j.sste.2018.11.006.
[25] M.Z. Ndii, Modelling the Use of Vaccine and Wolbachia on Dengue Transmission Dynamics, TropicalMed 5 (2020) 78. https://doi.org/10.3390/tropicalmed5020078.
[26] H. Zhu, S. Chen, W. Lu, K. Chen, Y. Feng, Z. Xie, Z. Zhang, L. Li, J. Ou, G. Chen, Study on the influence of meteorological factors on influenza in different regions and predictions based on an LSTM algorithm, BMC Public Health 22 (2022) 2335. https://doi.org/10.1186/s12889-022-14299-y.
[27] Z. Chen, Y. Liu, H. Yue, J. Chen, X. Hu, L. Zhou, B. Liang, G. Lin, P. Qin, W. Feng, D. Wang, D. Wu, The role of meteorological factors on influenza incidence among children in Guangzhou China, 2019-2022, Front Public Health 11 (2023) 1268073. https://doi.org/10.3389/fpubh.2023.1268073.

7. Supplementary figure and table

Supplementary Figure S1 : Meteorological conditions in New-Caledonia.

Panel A described the mean monthly precipitations measured in New Caledonia from January 2017To December 2023. **Panel B** described the daily precipitation over the study period measured from the meteorological station (blue line) and calculated from the satellite's assessment (purple line) using for representation a general additive method to smooth the lines from the ggplot package. Both lines were comparable, and consequently, no corrections were needed when using the satellite assessments. **Panel C** MEI index from January 2017 to December 2023: South Pacific oscillation bi-monthly Multivariate El Niño/Southern Oscillation (ENSO) index (MEI). The yellow dashed line represented the threshold for El Niño episodes and the red dashed line represented the thresholds for the La Niña episodes.

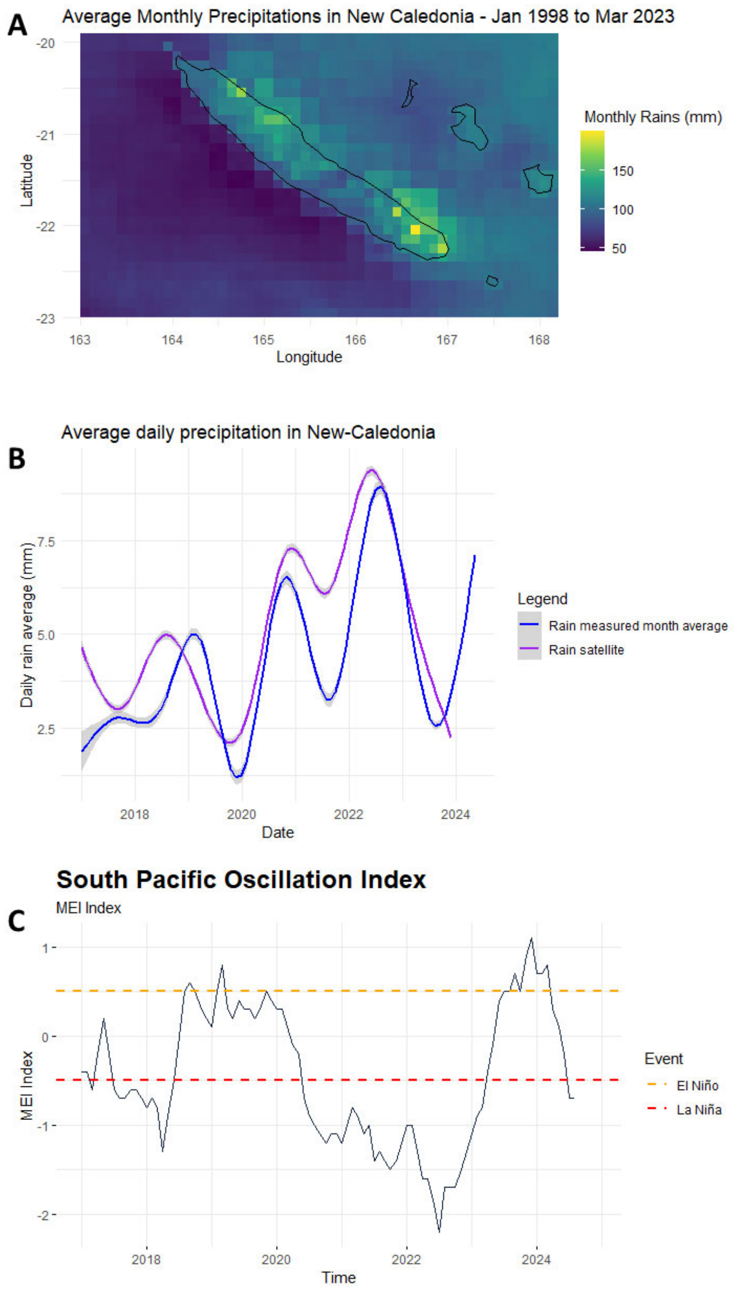

Supplementary Figure S2: Correlations between socio-economic variables – Principal Component Analysis (PCA)

Primary component analysis (PCA) of the 2019 census data performed as exploratory methods to assess the variables with the maximal variance between each communal district. The figure shows that the communal districts where people were more likely to drive a car and have a university degree (left side of the figure) had the opposite distribution to the commune where people were more likely to live in a tribe (right side of the figure).

The BEPC is the first level of education attained in the French education system. The percentage of inhabitants with at least this level of education is given.". The BAC represents the Baccalauréat, French high school diploma. The BEP and CAP are technical degrees obtained after BEPC without high school studies.

The percentage of people using a car (% car), public transports (% public transports) or walking (%walking is represented in the PCA.

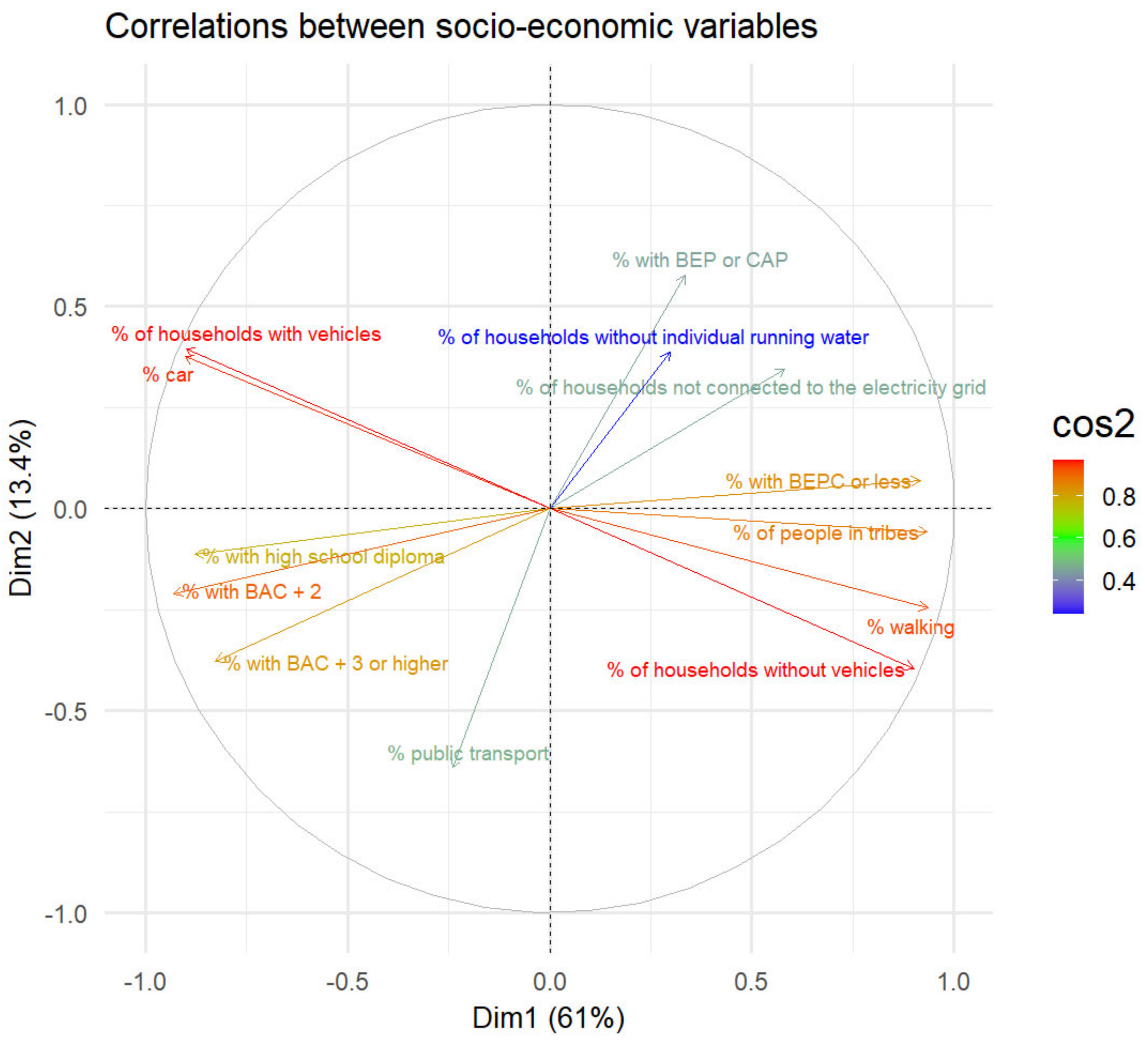

Supplementary Figure S3: Evolution of cases and moving 7 days averages of influenza, hepatitis A virus HAV, dengue and leptospirosis with their predicted models (orange) over the New-Caledonia.

Panel A, influenza cases biologically confirmed and the seven rolling day average of the cases (light blue). **Panel B,** Dengue (black), and *Wolbachia* was as well represented on the graph in marron. **Panel C,** Hepatitis A virus (HAV), the first cases were diagnosed in 2018 (Purple). **Panel D,** leptospirosis cases in red and the precipitations. Predicted models were described in orange.

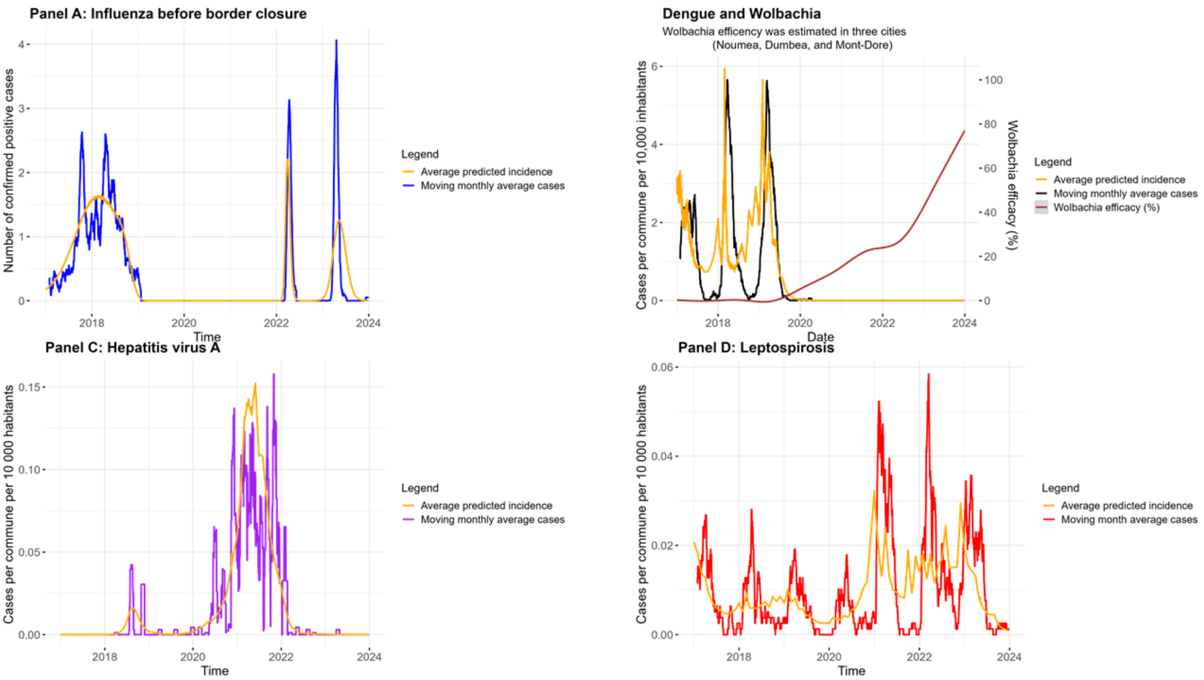

Supplementary Figure S4: dengue incidence per habitant and Wolbachia.

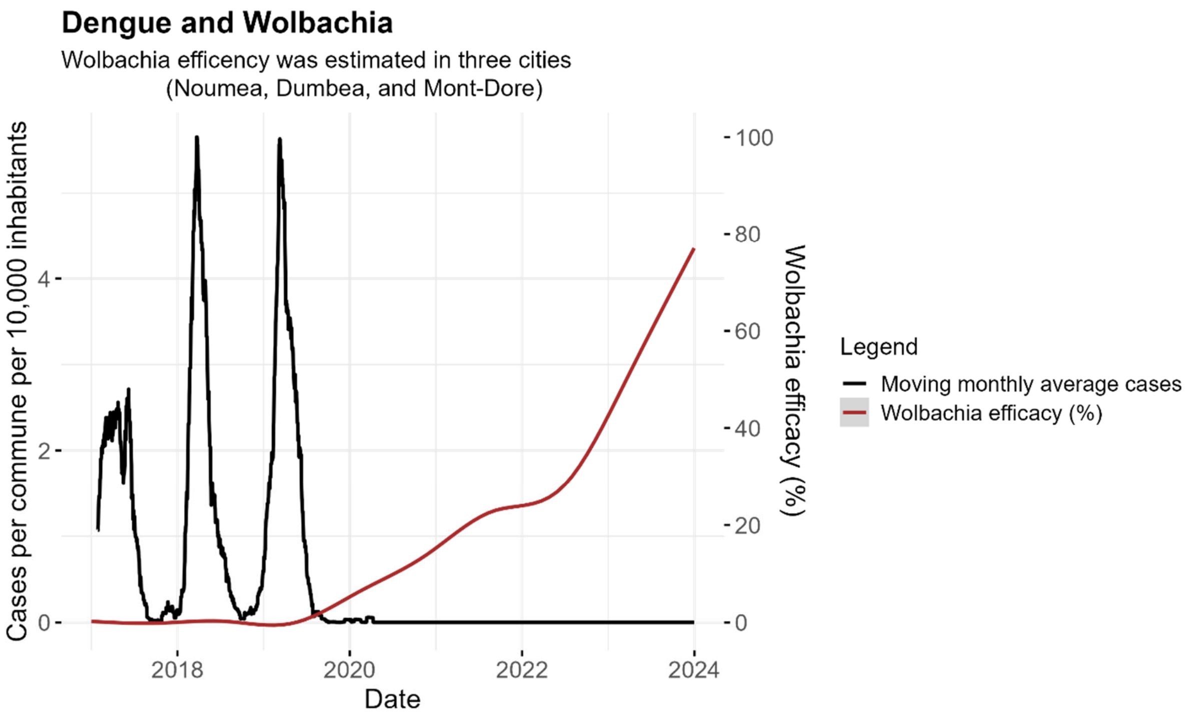

Supplementary Figure S5: cumulative effect of precipiration on leptospirosis using a GAMM method and a distributed lag non-linear model (DLNM) embedded

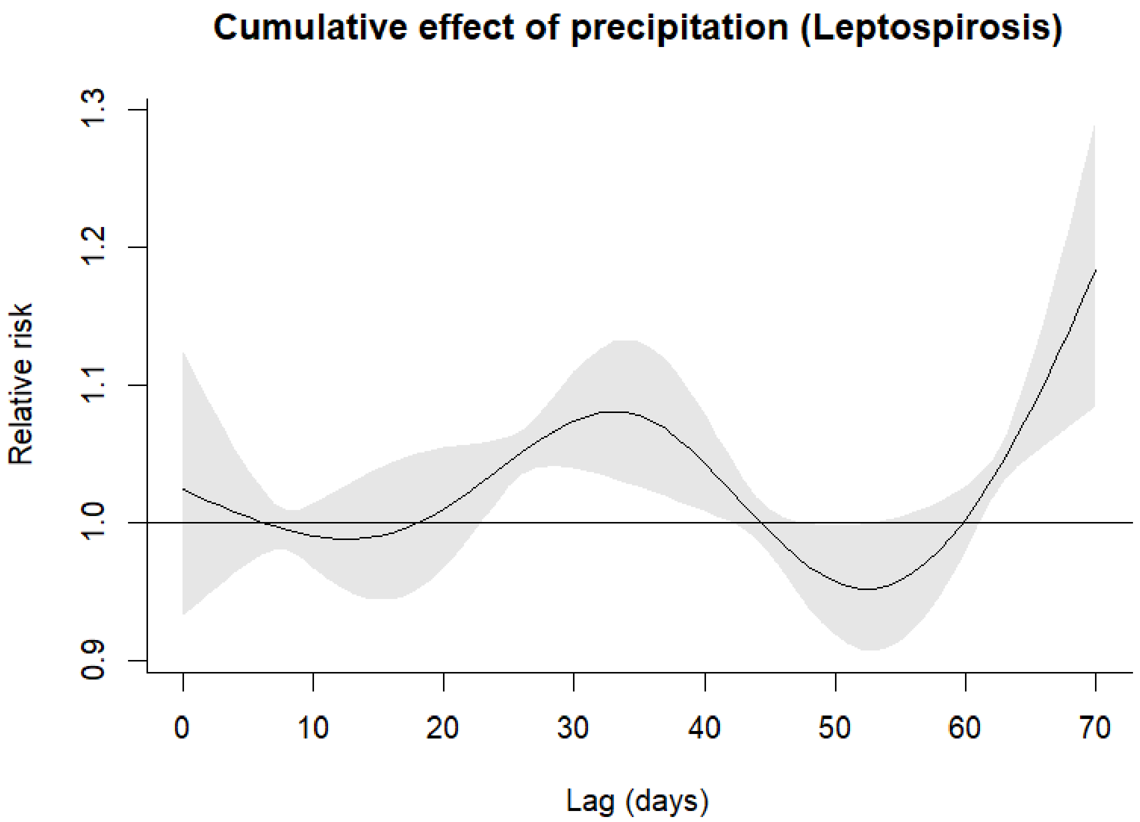

Supplementary Figure S6: Humidity and rain standardised values correlation represented through time

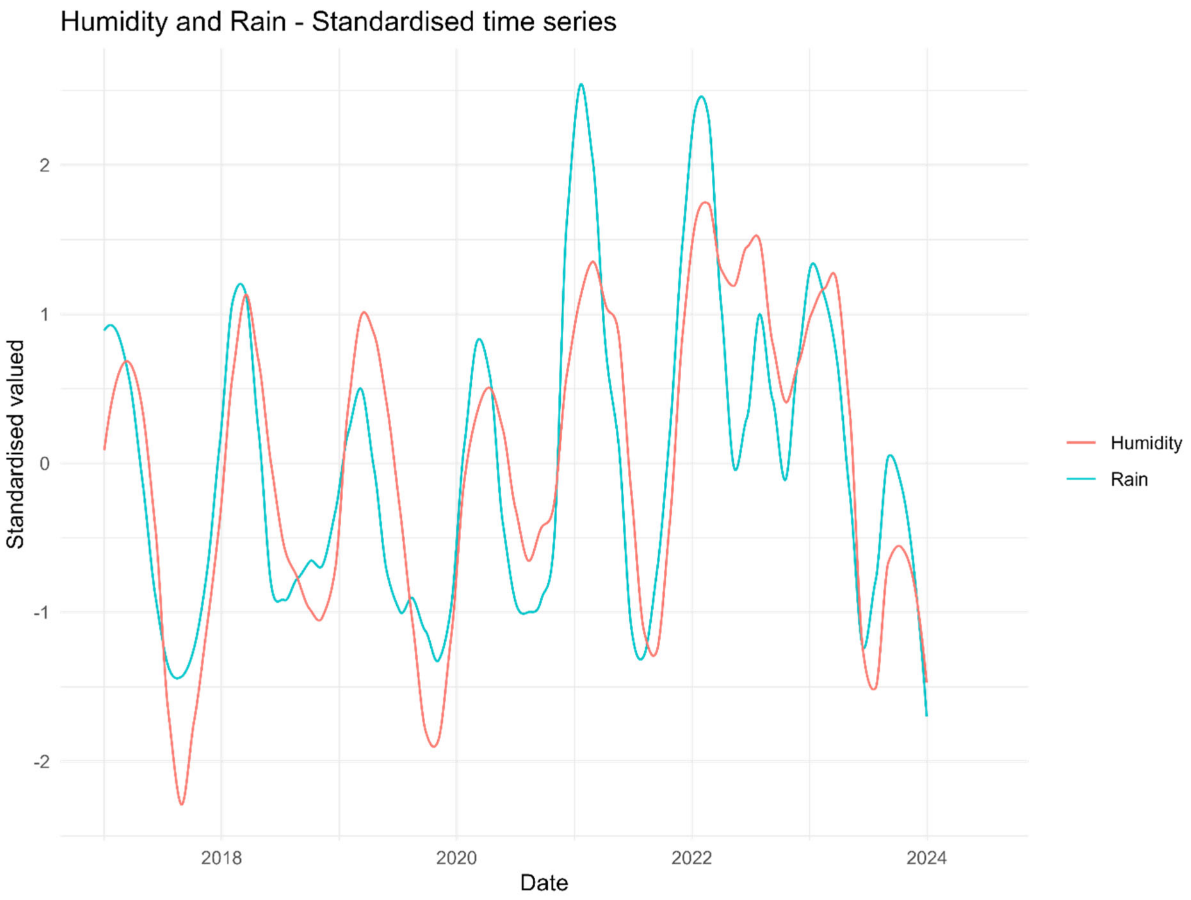

Supplementary Figure S7: Temperature and rain standardised values correlation represented through time

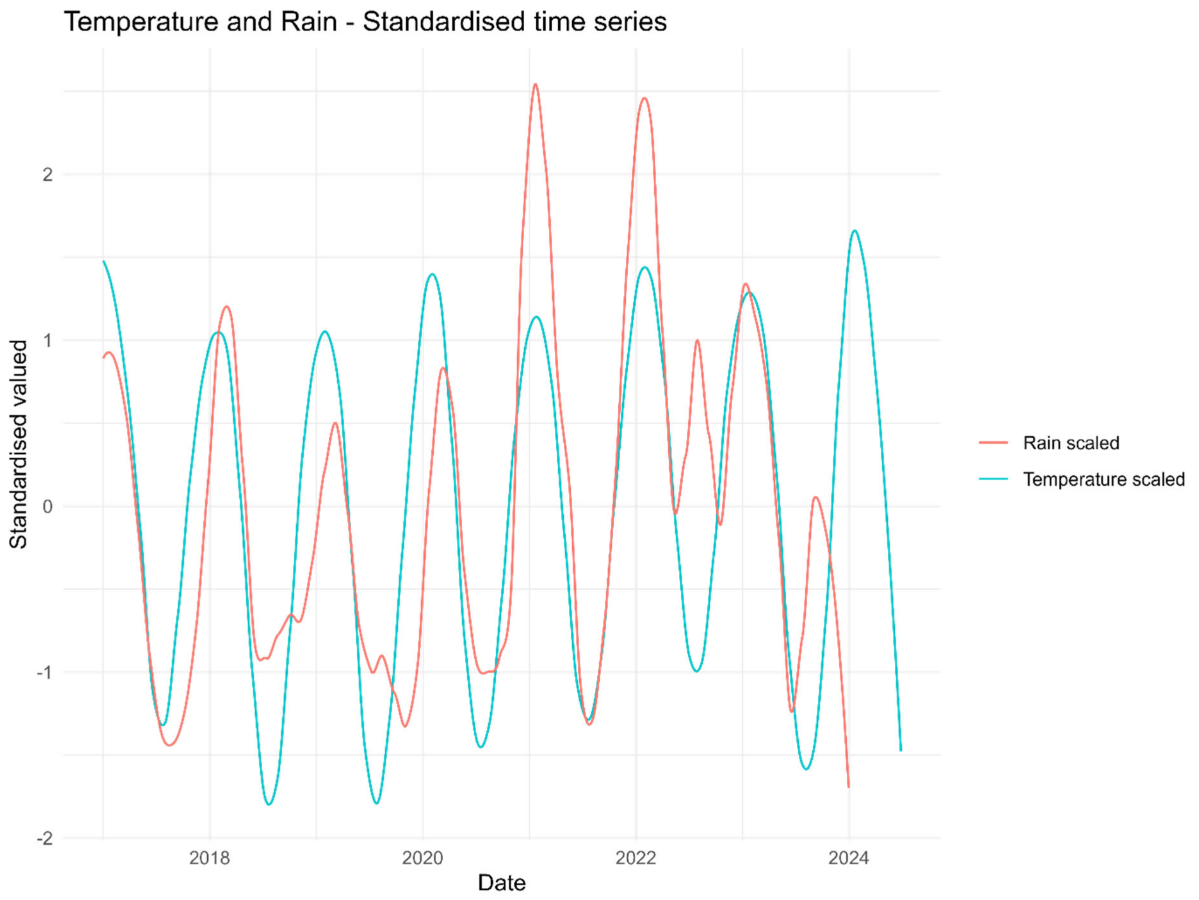

Supplementary Table S1: GAMM complete model for Influenza, Dengue, Hepatitis A virus (HAV), and leptospirosis with the complete variables of the formula MEI index and health care facilities.

The BEPC is the first level of education attained in the French education system. The percentage of inhabitants with at least this level of education is given."

	Influenza		Dengue		Hepatitis A virus (HAV)		Leptospirosis	
Variables	Odds Ratio 95% CI[12]	p-value[1]	Odds Ratio 95% CI[12]	p-value[1]	Odds Ratio 95% CI[12]	p-value[1]	Odds Ratio 95% CI[12]	p-value[1]
Mean daily precipitation over each commune (mm/d)	0.9 (0.7 - 1.1)	0.3	1.6 (1.4-2)	<0.001	1.7 (0.9-3)	0.09	1.2 (1 - 1.5)	<0.001
MEI index	0.4 (0.2- 0.7)	<0.001	0.5 0.4, 0.6	<0.001	3.62 (0.8- 17)	0.10	1.2 (0.8 - 1.8)	0.2
Number of health care facilities in each commune for 10 000 inhabitants	1.3 (1.1-1.6)	0.01	2.5 (1.4 - 4.4)	0.013	1.02 (0.9 - 1)	0.6	1.2 (1 - 1.5)	0.09
Percentage of inhabitants who walks as major transport mean	0.8 (0.6- 1.2)	0.4	0.7 (0.3- 1.8)	0.5	2.04 (0.2- 19)	0.5	1 (1 - 1)	0.06
Percentage of inhabitants in tribes	1 (1- 1)	0.2	1.04 (1.01- 1.08)	0.016	1.02 (0.3- 4)	>0.9	1.5 (1.01 - 2.2)	0.99
Percentage of inhabitants without water supply	1.2 (0.97- 1.5)	0.1	1 (0.6 -1.8)	0.9	0.89 (0.8-1.1)	0.2	0.7 (0.55 - 0.8)	0.003
BEPC	1 (1- 1)	0.6	1 (0.9- 1)	0.3	0.9 (0.7 -1.5)	0.2	1.1 (1 .1 - 1.1)	0.001
s(Date)		<0.001		<0.001		<0.001		<0.001
s(Communal distric)		<0.001		<0.001		<0.001		<0.001

Note: BEPC: first grade diploma obtained at 14-15years old. Odds ratios were calculated based on the exponent of the coefficient. CI: Confidence Interval; MEI : Multivariate El Niño/Southern Oscillation (ENSO) index, s: spline; NA: Non applicable
